# The Development and Application of Airway Devices in China

**Published:** 2016-10-01

**Authors:** Xiangdong Chen, Wuhua Ma, Renyu Liu, Shanglong Yao

**Affiliations:** 1 Department of Anesthesiology, Institute of Anesthesiology and Critical Care Medicine, Union Hospital of Tongji Medical College, Huazhong University of Science and Technology, Wuhan, China; 2 The First Affiliated Hospital of Guangzhou University of Chinese Medicine, GuangZhou, China; 3 Department of Anesthesiology and Critical Care at the Hospital of the University of Pennsylvania, Pennsylvania, USA

**Keywords:** Development Application, Airway devices, Airway management, China

## Abstract

Airway management is one of the most important tasks for anesthesiologists. Anesthesiologists are experts in airway management and have made tremendous contribution to the development of the airway devices. Chinese anesthesiologists have made significant contribution in introducing advanced airway management and developing innovative techniques and devices for airway management in China. This article overviews the development and application of airway devices in China as well as the dedication and contribution of Chinese experts in the development of novel airway devices. With the development of science and technology accompanied by the advanced knowledge in airway management, more effective and safe artificial airways will be developed for clinical practice. The authors believe that Chinese experts will continue their outstanding contribution to the development of innovative airway devices, systems and knowledge.

## Introduction

Tuo Hua, a well-known ancient Chinese physician demonstrated the use of one anesthetic named “Ma Fei San” to provide anesthesia about 1800 years ago,[1] while anesthesia was not available to the public domain until Crawford Long used ether for the first time on March 30, 1842 and William T.G. Morton administered ether anesthesia at Massachusetts General Hospital on October 16,1846.[2] At the same time, respiratory depression from anesthesia was observed as a potential fatal side effect.[3] While scientists continue to develop safe anesthetics with minimal side effects,[4] control of the patient’s respiration and airway is needed in most occasions during general anesthesia. Thus, airway management is always a key topic in clinical anesthesia.[5]

Maintenance of effective ventilation and oxygenation is essential for anesthesiologists.[6] Anesthesiologists manage the breathing of patients during general anesthesia.[7] Artificial airway devices help doctors to manage the airway, maintain a clear airway and proper oxygenation.

The history of airway devices in China can traced back to Ming dynasty in a famous book named Pu-Ji-Fang. At that time, doctors maintained the breathing of patients by a reed tube similar to a supraglottic airway. Until 1950s, anesthesiologists who studied abroad introduced simple tracheal tubes consisting of a rubbery tube and a homemade airbag into China. Since then, many modern airway devices and airway auxiliary devices, such as laryngeal mask airway, video laryngoscope, and fiber bronchoscope and so on, were introduced or invented. The development of airway devices has made dramatic progress in the past 100 years.[8] This article chronicles the development and application of airway devices in China and overviews the dedication of Chinese doctors to the development of airway devices.

### 1. The development of airway instrument

#### 1.1. Development of tracheal tube

**Figure 1:**
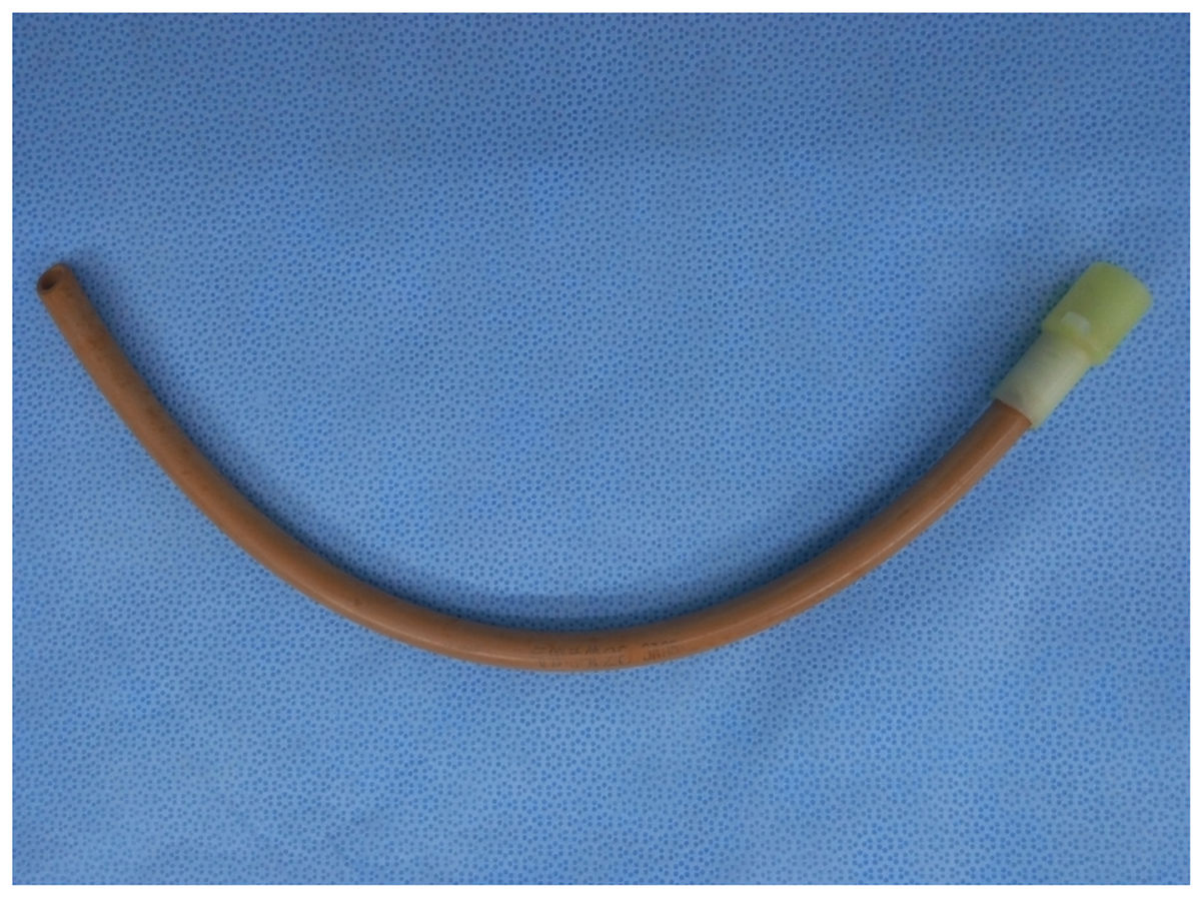
Rubber tracheal tube.

**Figure 2:**
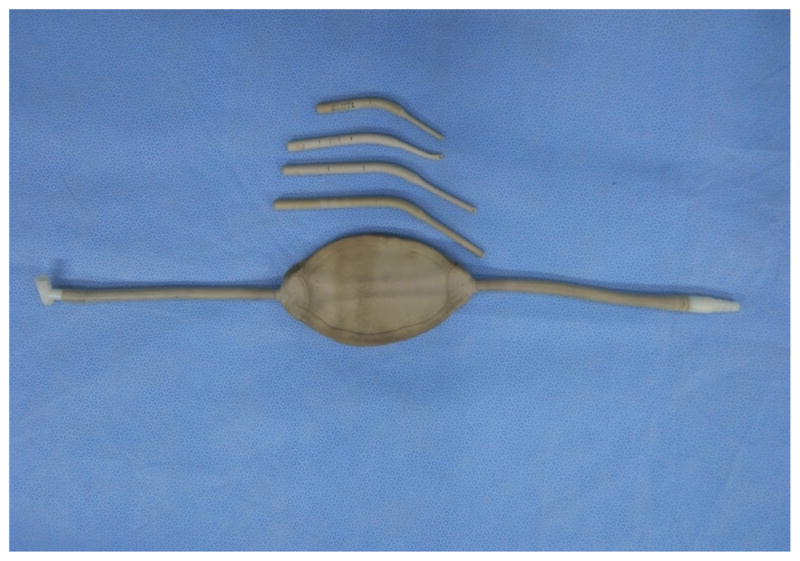
Breath bag for neonatal child

**Figure 3:**
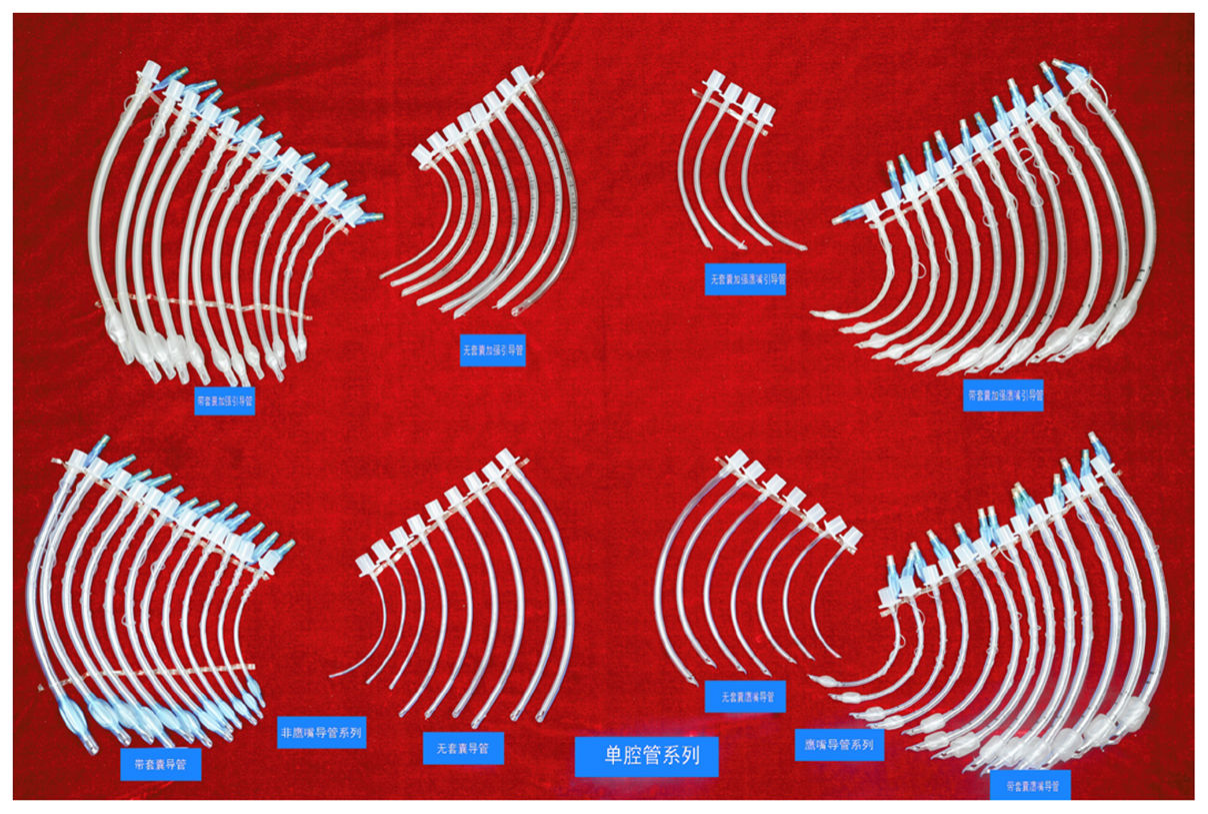
Various tracheal tube

**Figure 4:**
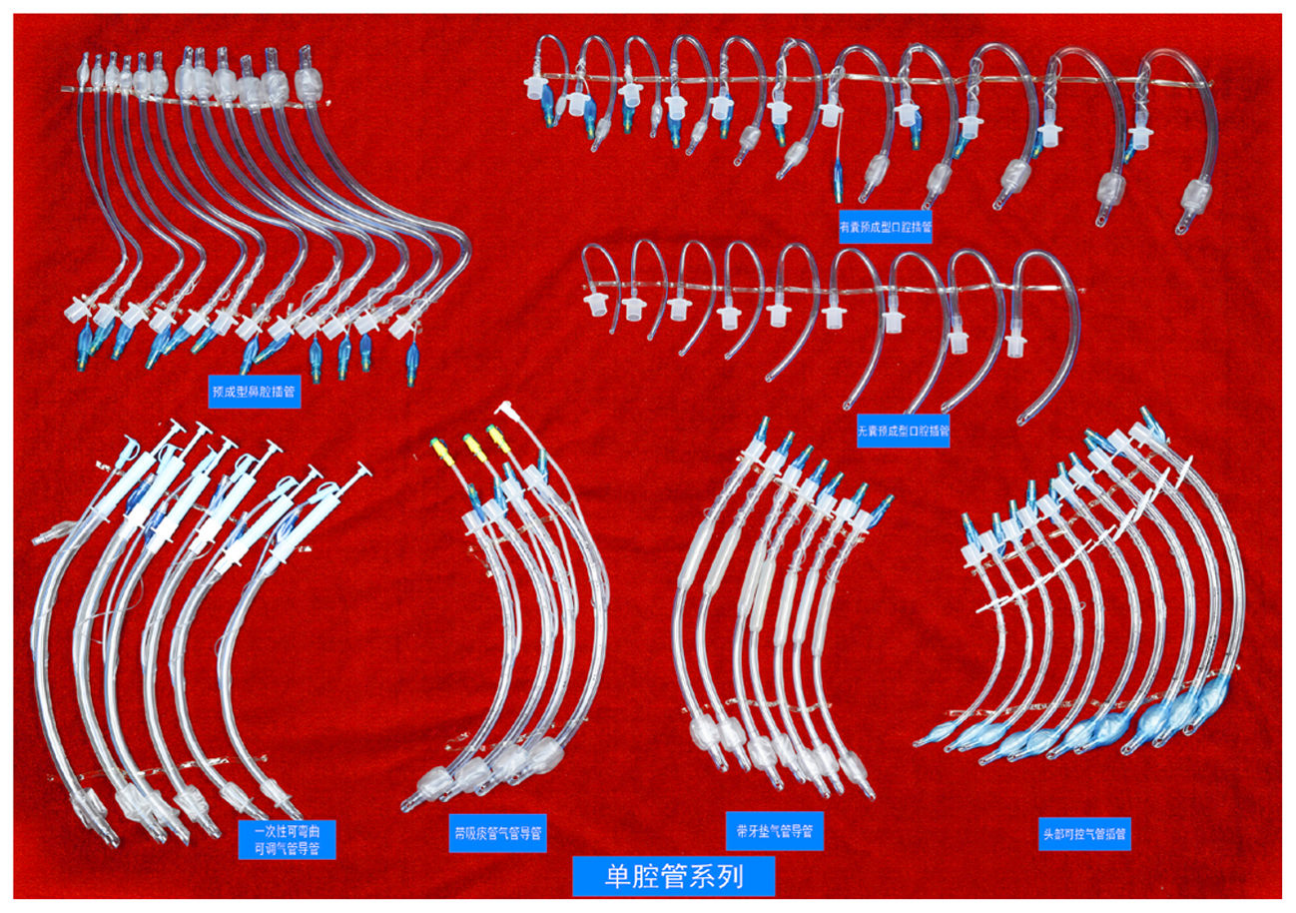
Special tracheal tube

**Figure 5:**
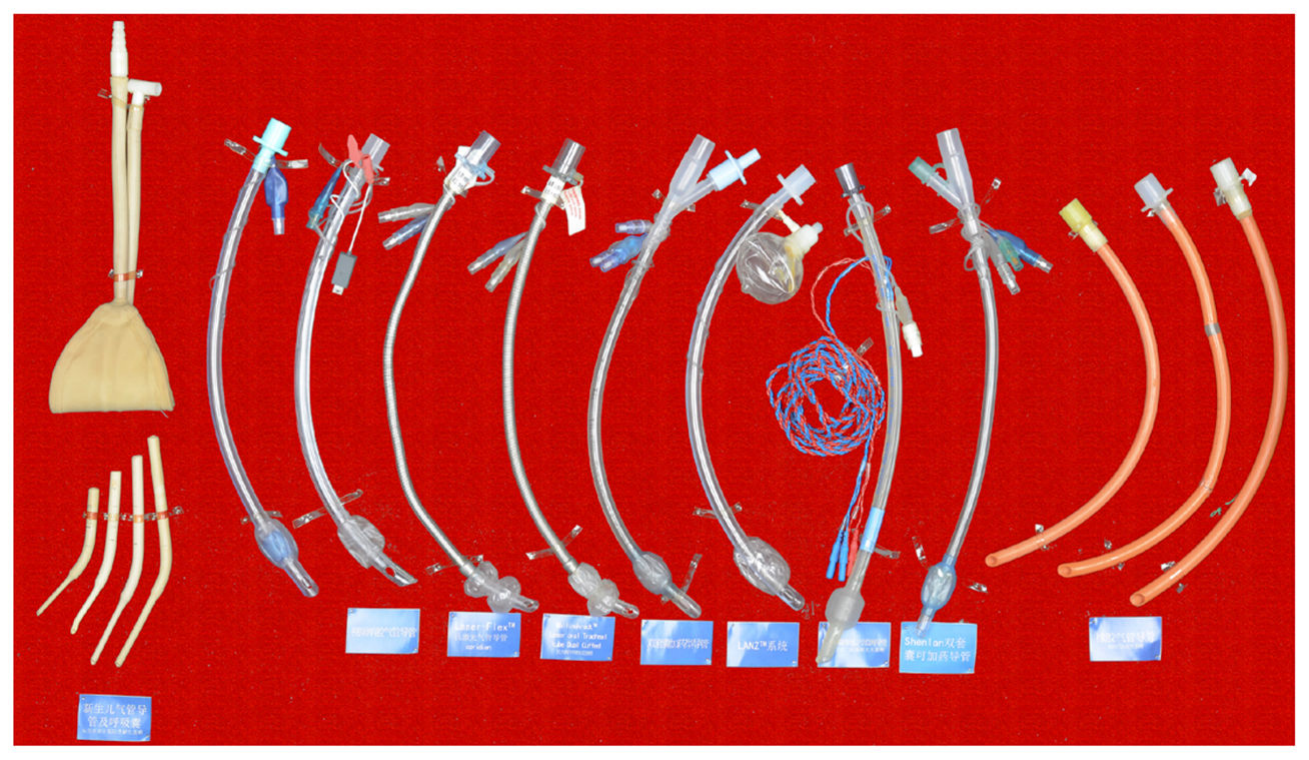
Laser tube, et al.

Dr. Trendelenburg, a German physician invented an inflatable cuff catheter in 1871; the Magill rubber tracheal catheter was used in clinical anesthesia in 1917 [9]. The cuffed tracheal tube appeared in 1950s that combined a rubber tube and a self-made cuff (Figure 1) followed by the tracheal tube and breathing bag for neonates (Figure 2). The early tracheal tubes used in China consisted of a simple rubber tube with homemade balloon, introduced by some Chinese anesthesiologists trained abroad around 1950s. Since then, there are many different kinds of tracheal tubes such as the simple tube, reinforced tube, and laser-resistant tube etc. (Figure 3–5).

**Figure 6:**
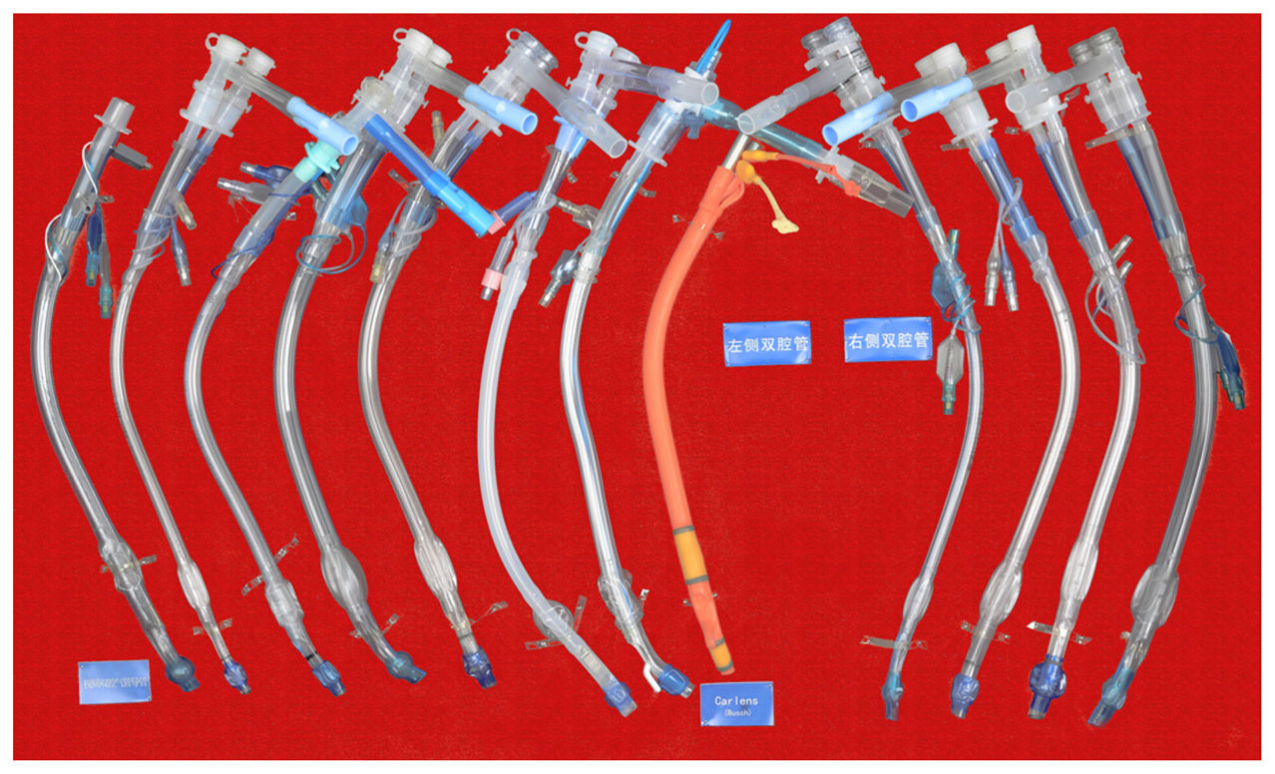
Double-lumen tube.

**Figure 7:**
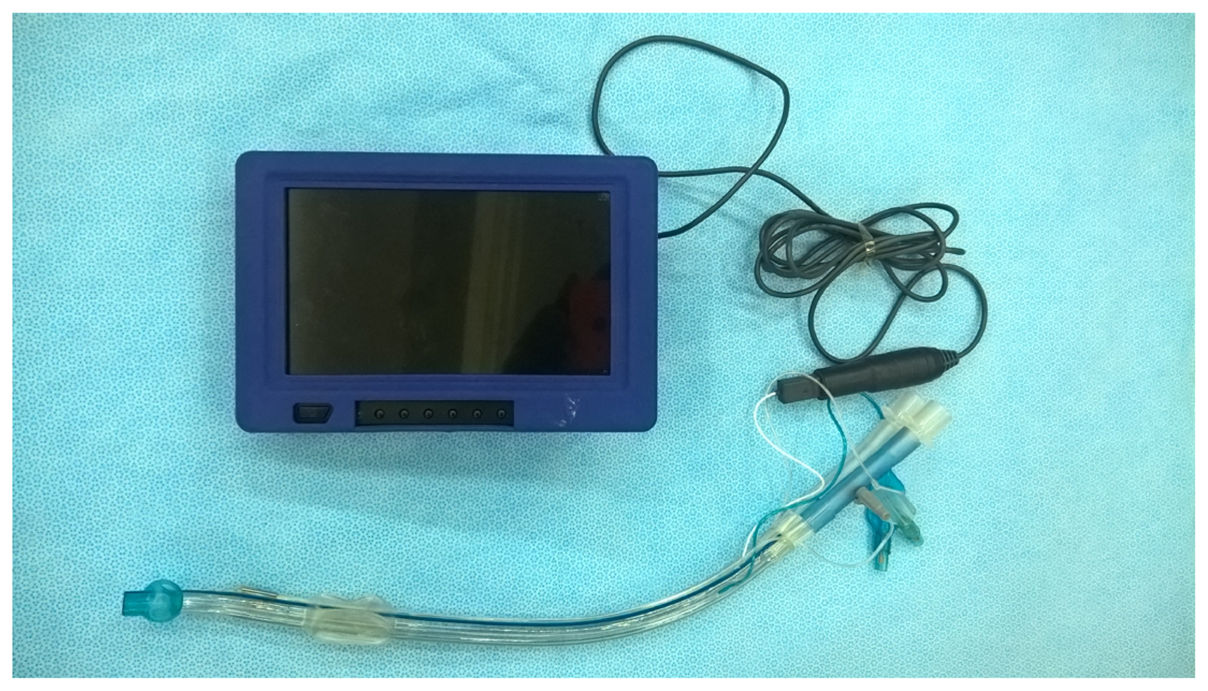
Visual double-lumen tube.

To meet the requirement for thoracic surgery, the double-lumen tube was invented. There are also several kinds of double-lumen tube such as Robert Show tube and Carlens tube. The visual double-lumen tube invented by Chinese anesthesiologist appeared in 2014, an innovative effort for the fast and precision positioning of double-lumen tube (Figure 6, 7).

#### 1.2. Development of the laryngoscope

A laryngoscope is an important auxiliary instrument for intubation, from early Macintosh and Miller laryngoscope blades to an adjustable laryngoscope. The birth of video laryngoscopy [10] indicated that the application of visual technology had resulted in a breakthrough in medicine.

**Figure 8:**
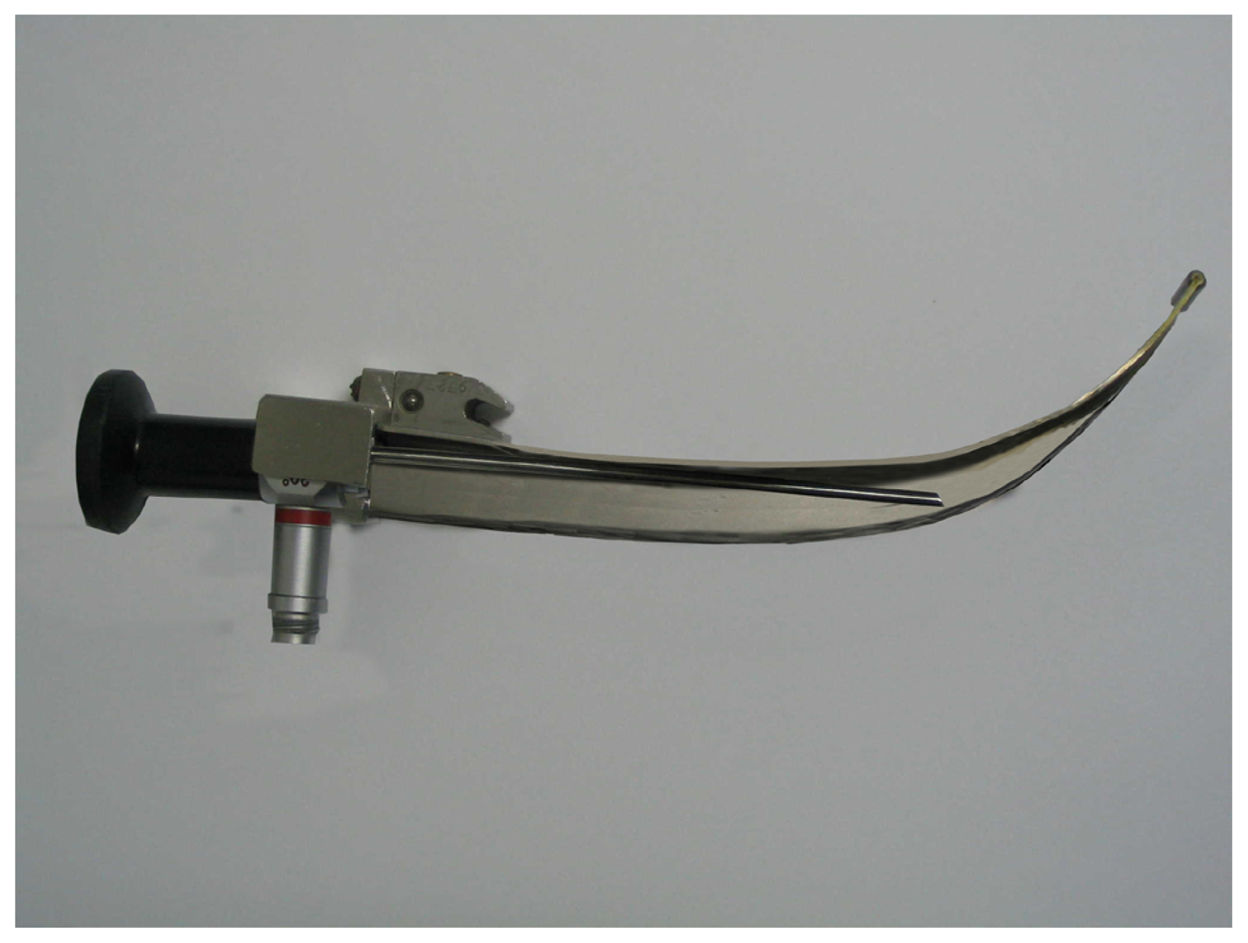
WH-A fiber optic laryngoscope.

**Figure 9:**
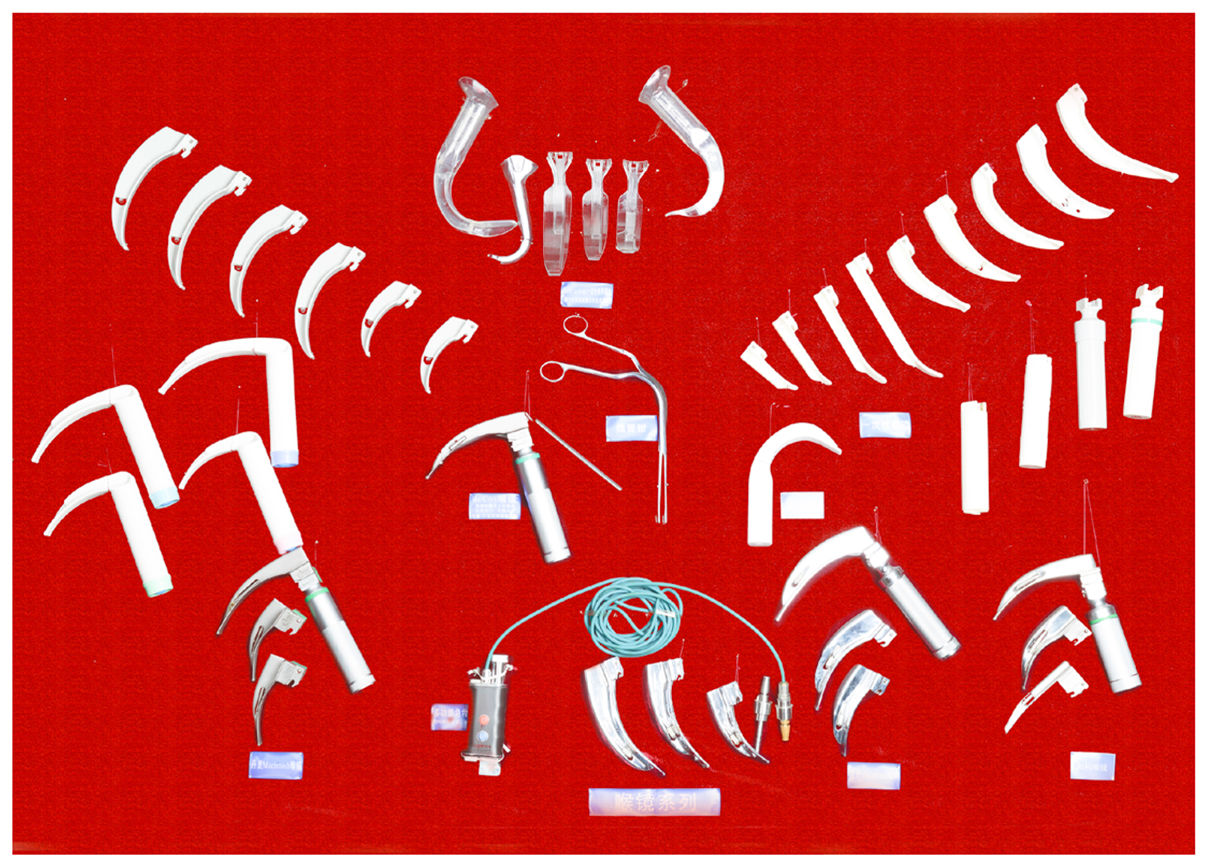
Traditional laryngoscope.

**Figure 10:**
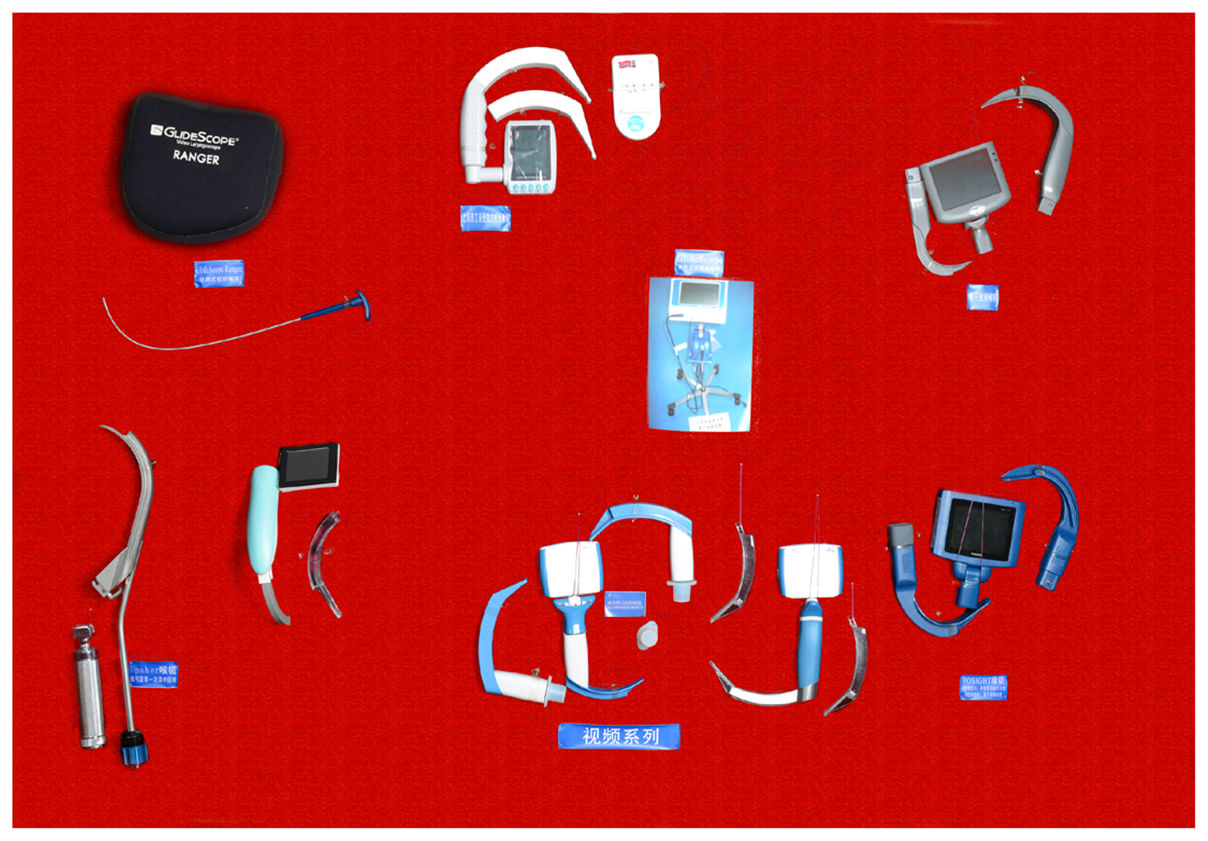
Video laryngoscope.

**Figure 11:**
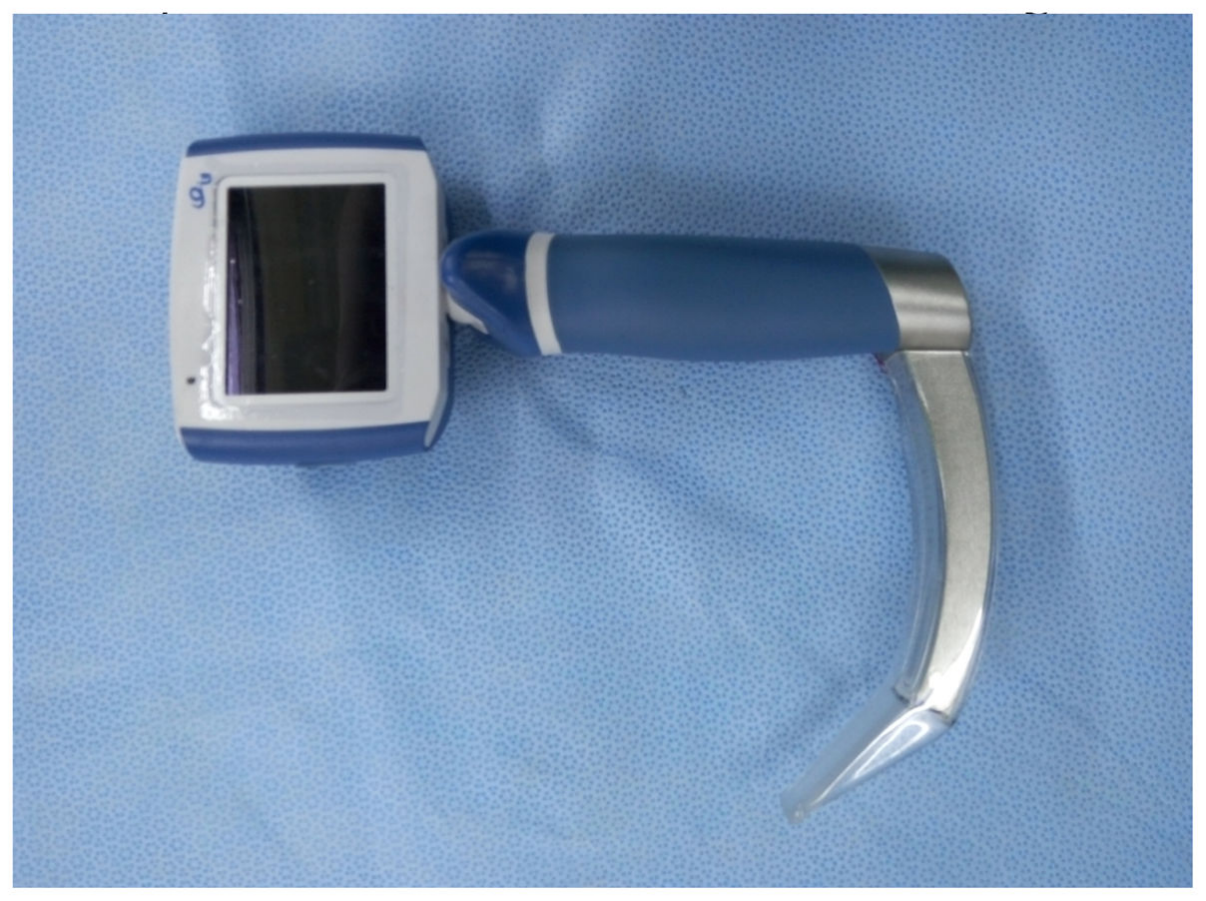
UE video laryngoscope.

Video laryngoscopy now plays an important role in intubation especially in the management of difficult airways in China, which takes the use of technology of intubation to a safer level. Moreover, the intubation technique using video laryngoscopy was added to the ‘Guideline of difficult airway management of Chinese Society of Anesthesiology. To tackle the difficult airway, Professor Shang-long Yao from Wuhan Union Hospital was enthusiastically developing WH-A fiber optic laryngoscope since 1987 (Figure 8), which was considered as the first generation of video laryngoscopes in China. After that, different kinds of laryngoscopes have been developed, such as the UE video laryngoscope, the TOSIGHT video laryngoscope and the insight laryngoscope (Figure 9, 10). The UE video laryngoscope that was invented by Professor Shang-long Yao and Professor Fu-shan Xue, based on Chinese airway anatomy, and won the first prize for scientific and technological progress of Hubei province recently (Figure 11). UE video laryngoscopes are now exported to more than 20 countries and regions.

#### 1.3. Development of the bronchial-blocking tube

**Figure 12:**
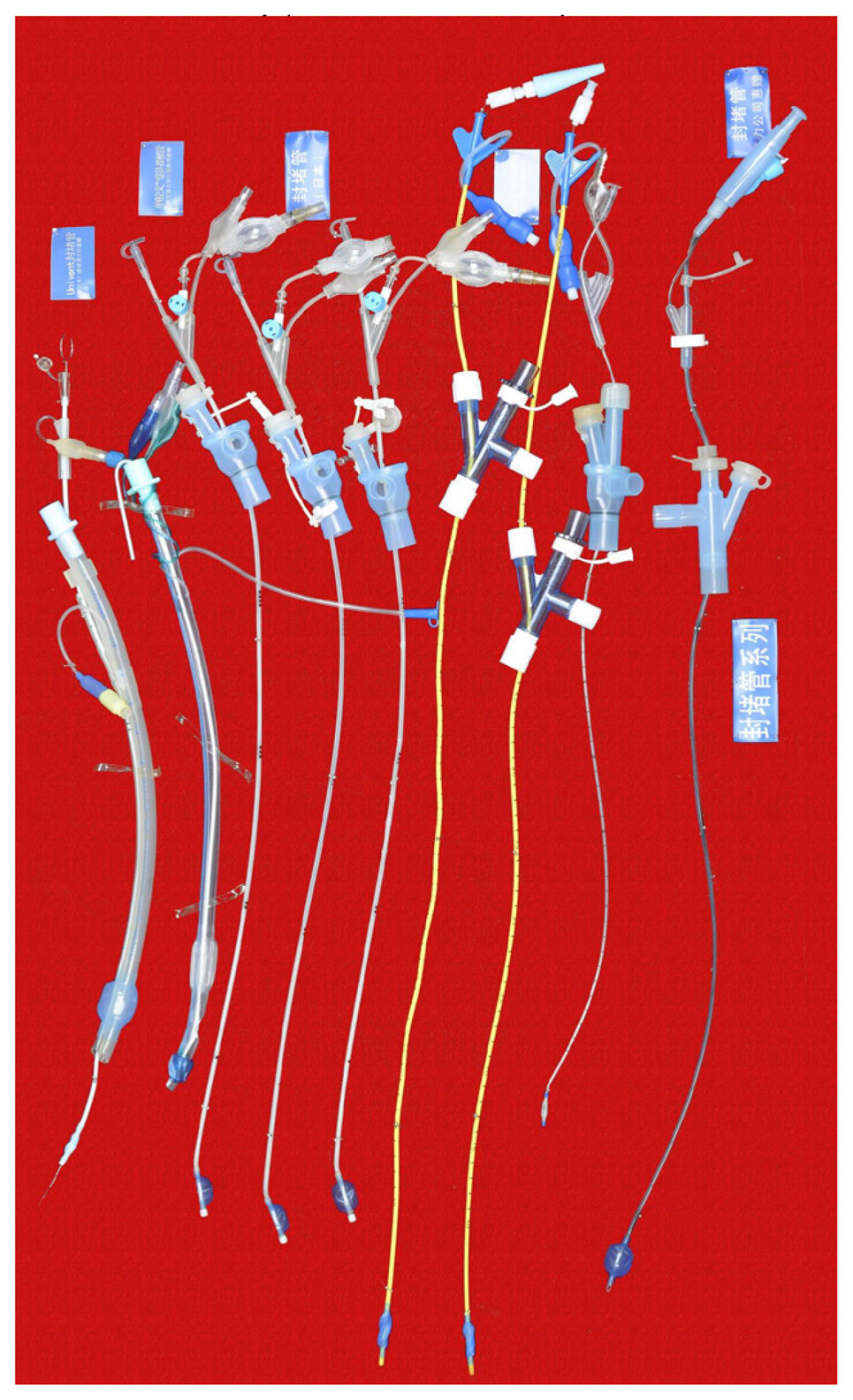
Bronchial-blocking tube.

**Figure 13:**
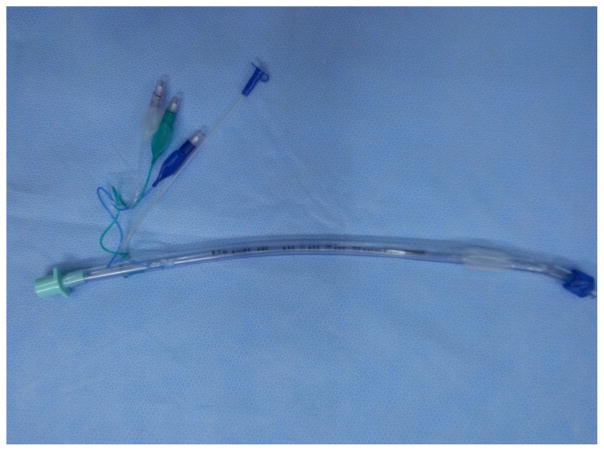
Single-lumen blocking tube.

Bronchial-blocking tubes have had a large success in recent years and are likely to become novel supplements for the pulmonary sequestration technique. Chinese anesthesiologists usually use bronchial-blocking tubes on the base of simple tracheal tubes (Figure 12). Professor Wei Jiang invented the single lumen bronchial-blocking tubes which are used when the double-lumen tubes’ lumen are too large for Chinese patients (Figure 13).

#### 1.4. Development of the laryngeal mask airway (LMA)

LMA is the most commonly used supraglottic airway in China. The advantages of using LMA for airway management includes less injury and stimulation, easy usage, and no need of muscle relaxant.

**Figure 14:**
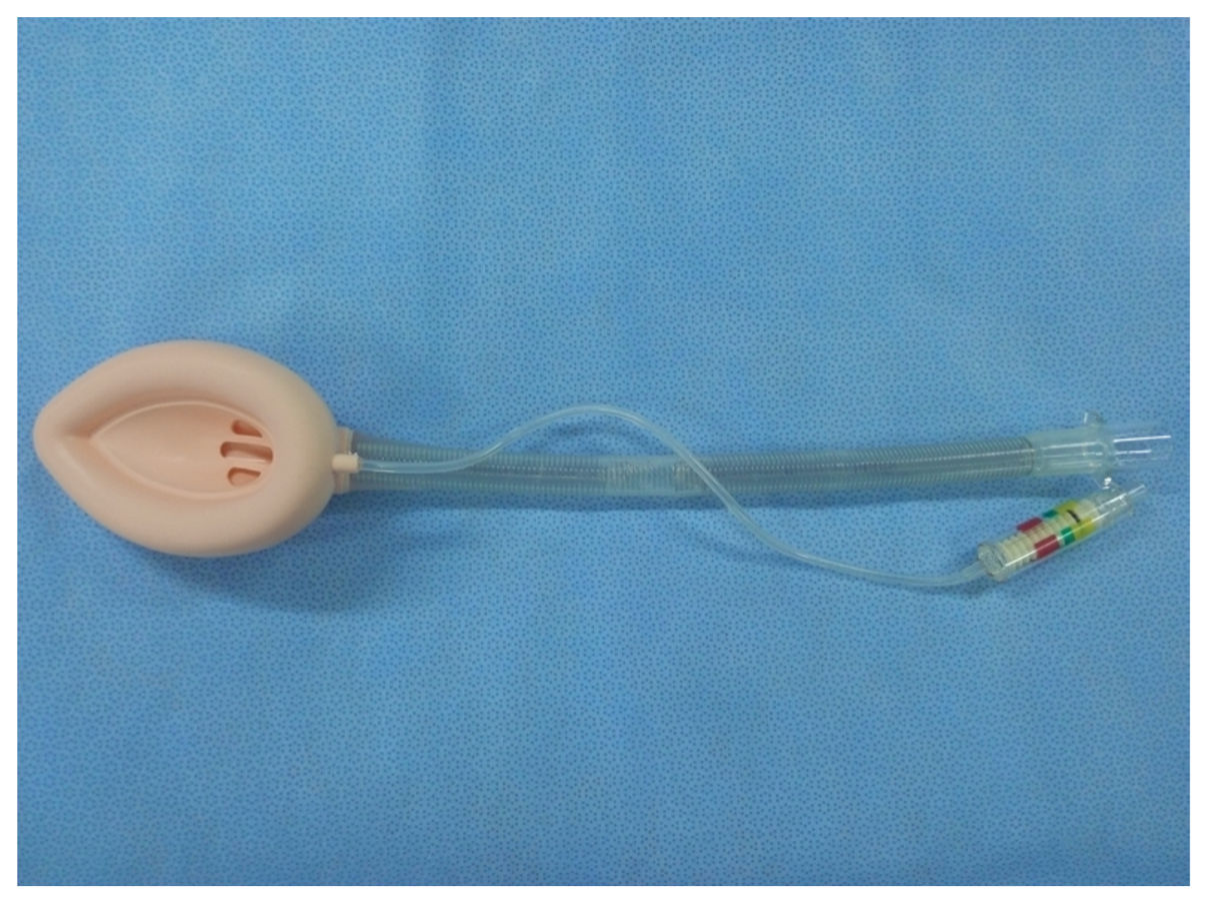
Medis LMA.

**Figure 15:**
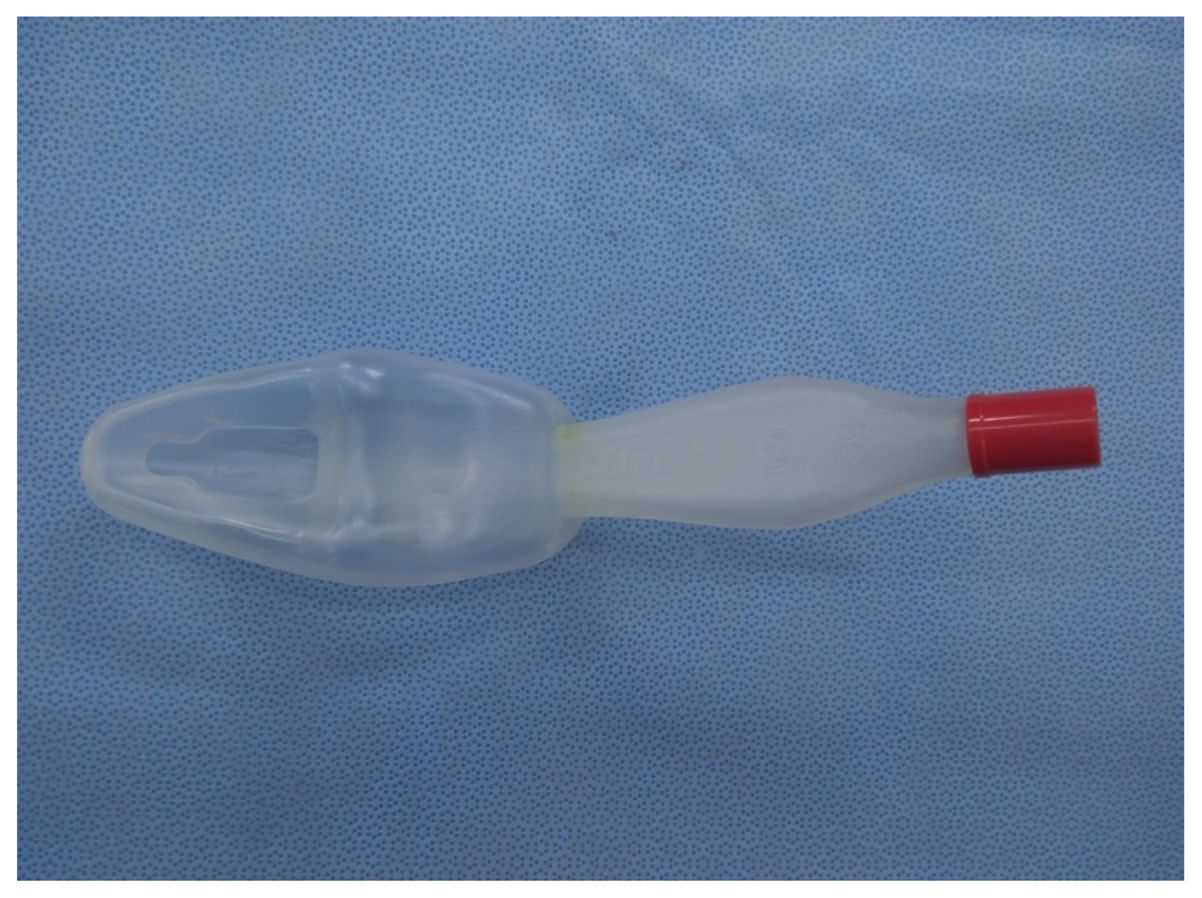
Slipa LMAFigure.

**Figure 16:**
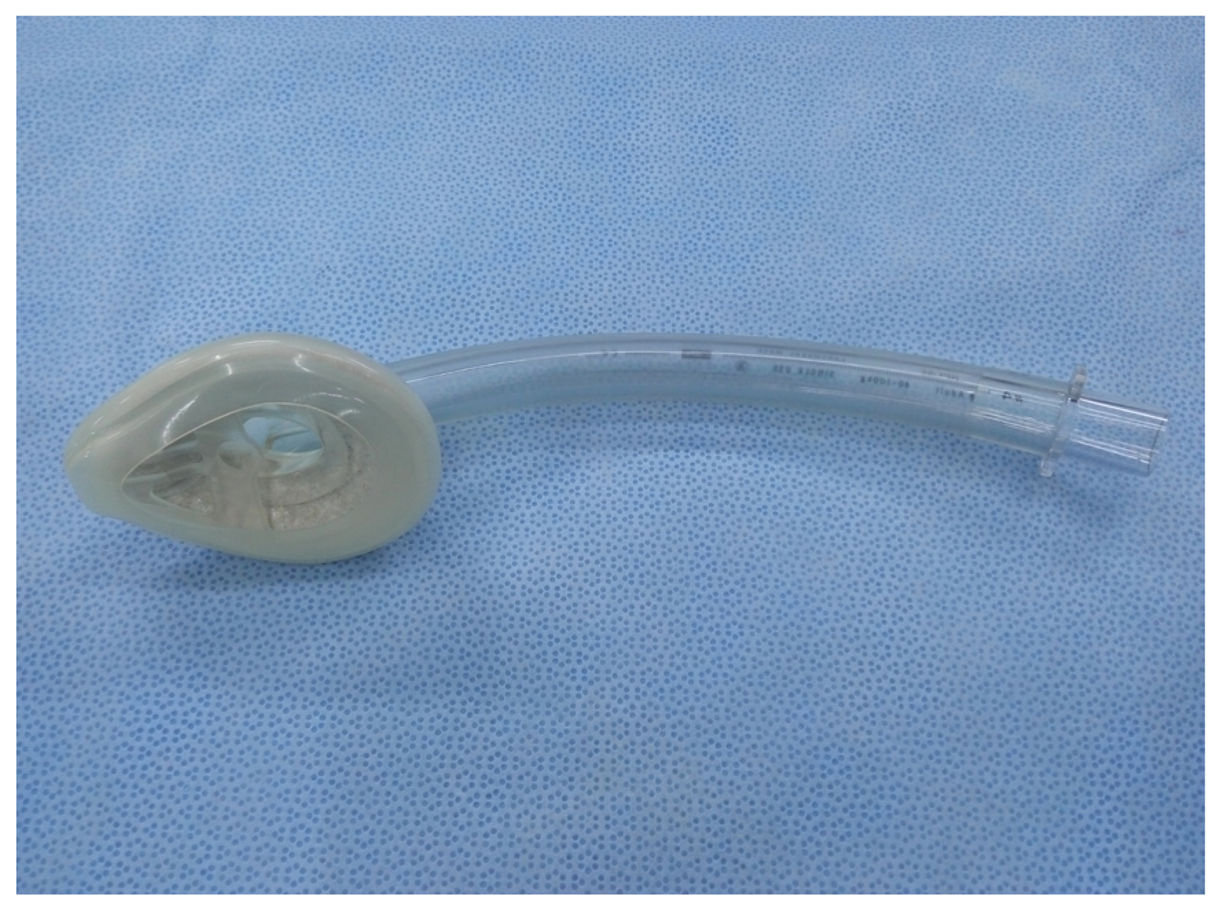
Oplac LMA.

**Figure 17:**
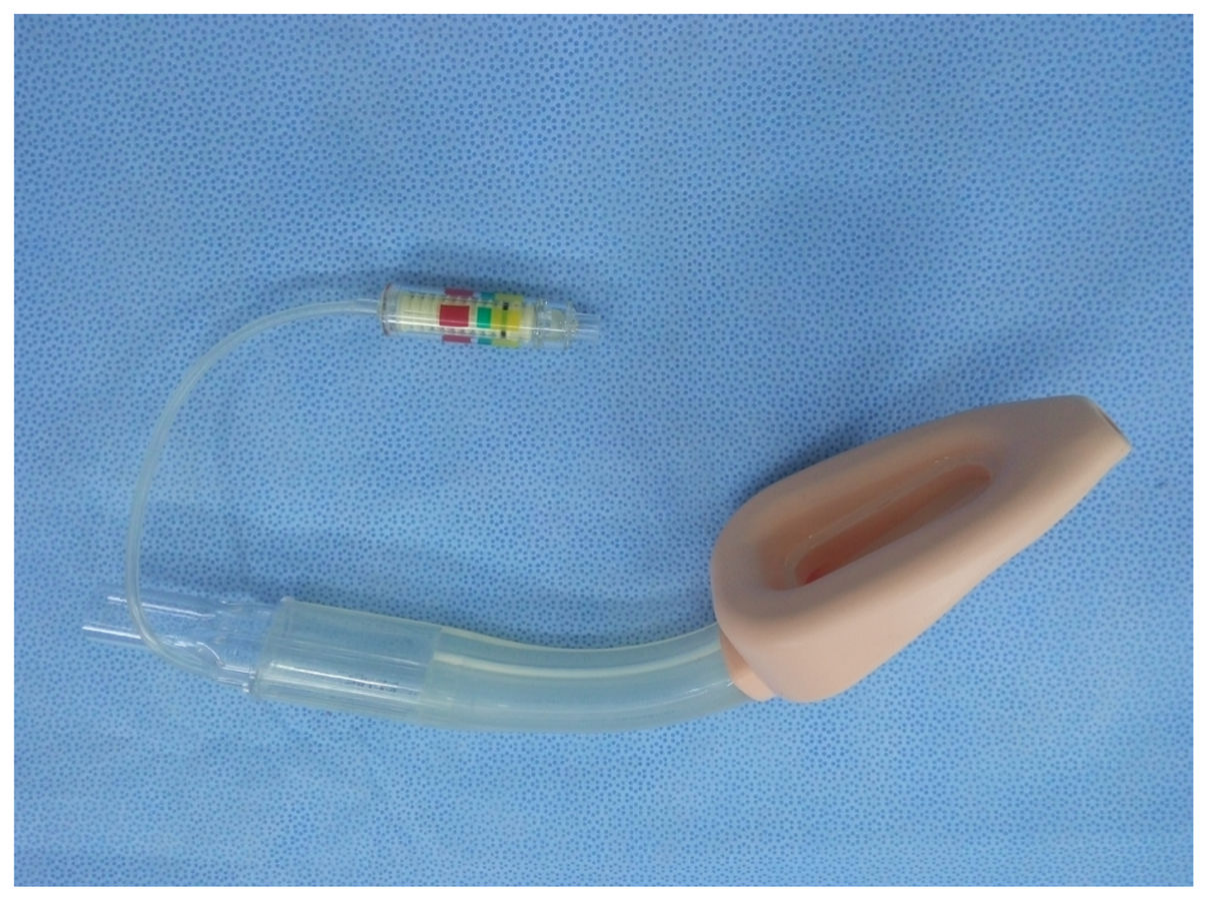
Ming-renfastrach LMA

**Figure 18:**
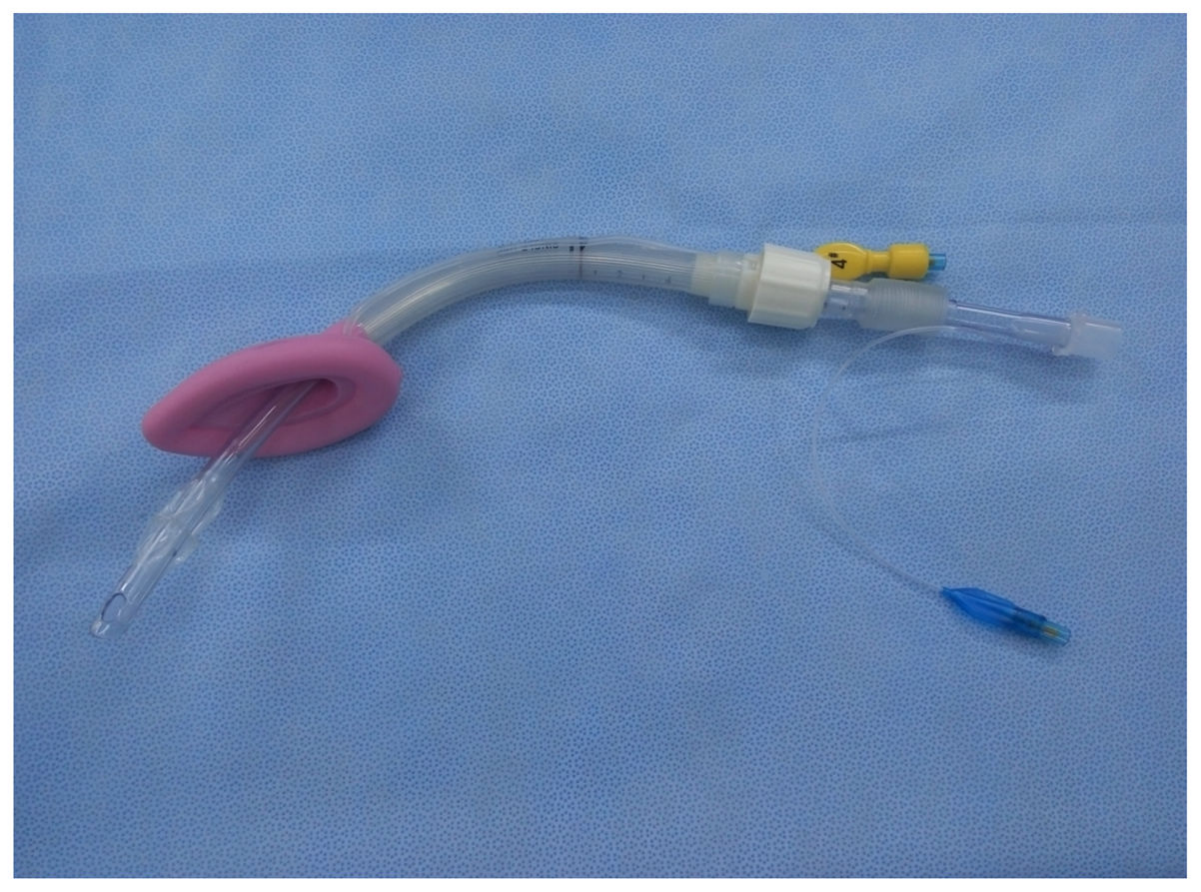
Intubating LMA

Professor Ming-Zhang Zuo has made significant contributions in promoting the usage of LMA since its introduction in China. Chinese companies have also made important modifications during the manufacture of LMA for various purposes such as Medis LMA (Figure 14). Many kinds of inflatable LMA are available in the market including Slipa LMA manufactured in Hangzhou (Figure 15) and Oplac LMA (Figure 16) manufactured in Taiwan. Ming-renfastrach LMA (Figure 17) invented by Professor Ming Tian and the endotracheal tube LMA (Figure 18) invented by Professor Jin-Fang Xiao will be in the market soon. A novel visual LMA invented by professor Wu-hua Ma will appear in clinical practice in the near future.

#### 1.5. Development of the light wand and visual stylet

**Figure 19:**
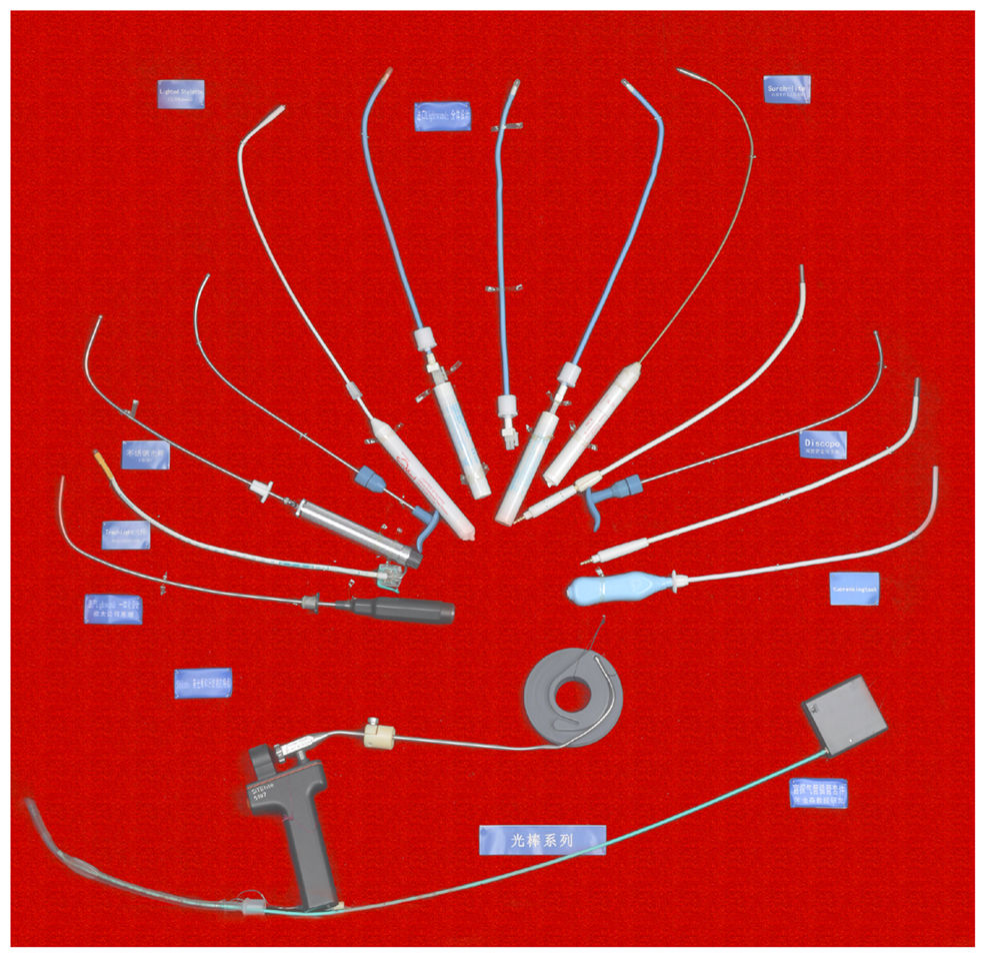
Optical stylets.

The development of the laryngoscope progressed from blind to video-guided instrument, and the development of the light wand in China also progressed from a self-made light wand to the visual stylet. Professor Ye-sen Zhu first invented the blind tracheal intubation kit, and subsequently, a large number of new visual wands and stylets appeared one after another, such as the blind light wand invented by Shanghai VEDIO company; Disposcope invented in Taiwan and the all-in-one light wand invented by MDH, Youyi and VEDIO companies (Figure 19).

#### 1.6. Development of the Supraglottic airway

An esophageal-tracheal combi-tube [12] is a kind of emergency airway device that combines the esophageal-blocking function as well as the tracheal ventilation function as a supraglottic airway. It is usually used to tackle with difficult ventilation and difficult intubation during emergency. Similar to esophageal-tracheal combi-tube, the laryngeal tube [13] is a kind of single-lumen and two-cuff artificial airway that is sometimes used in emergency airway; however, it may not prevent regurgitation of gastric content and aspiration. The Cobra perilaryngeal airway [14] is a disposable supraglottic airway intended for use in spontaneously and mechanically ventilated patients.

#### 1.7. Development of subglottic emergency airway

Thyrocricoid puncture kit, intercricothyrotomy kit, and tracheotomy kits invented in other countries are very popular in China. The Chinese Weili company also invented and manufactured a manual jet ventilation device and a tracheotomy kit in 2014.

#### 1.8. Development of the mask

There are many different kinds of masks which have appeared in clinical practice, from the early opaque mask to the transparent mask, from the oxygen mask to the nebulizer mask, from the inflatable mask to the non-inflatable mask and so on.

#### 1.9. Fiber bronchoscope

Fiber bronchoscope was first used in clinic during late 1960s and early 1970s. The difficult airway guideline published by ASA promoted the utilization of video laryngoscope in airway management, and fiber optic bronchoscopy tracheal intubation is now the gold standard of noninvasive intubation. [15] Fiberoptic bronchoscopy tracheal intubation is an important tool for difficult airway management in various hospitals in China. [16] The training of fiberoptic bronchoscopy tracheal intubation is a regular subject in teaching hospitals.

#### 1.10. Development of the visual flexible bronchoscope

**Figure 20:**
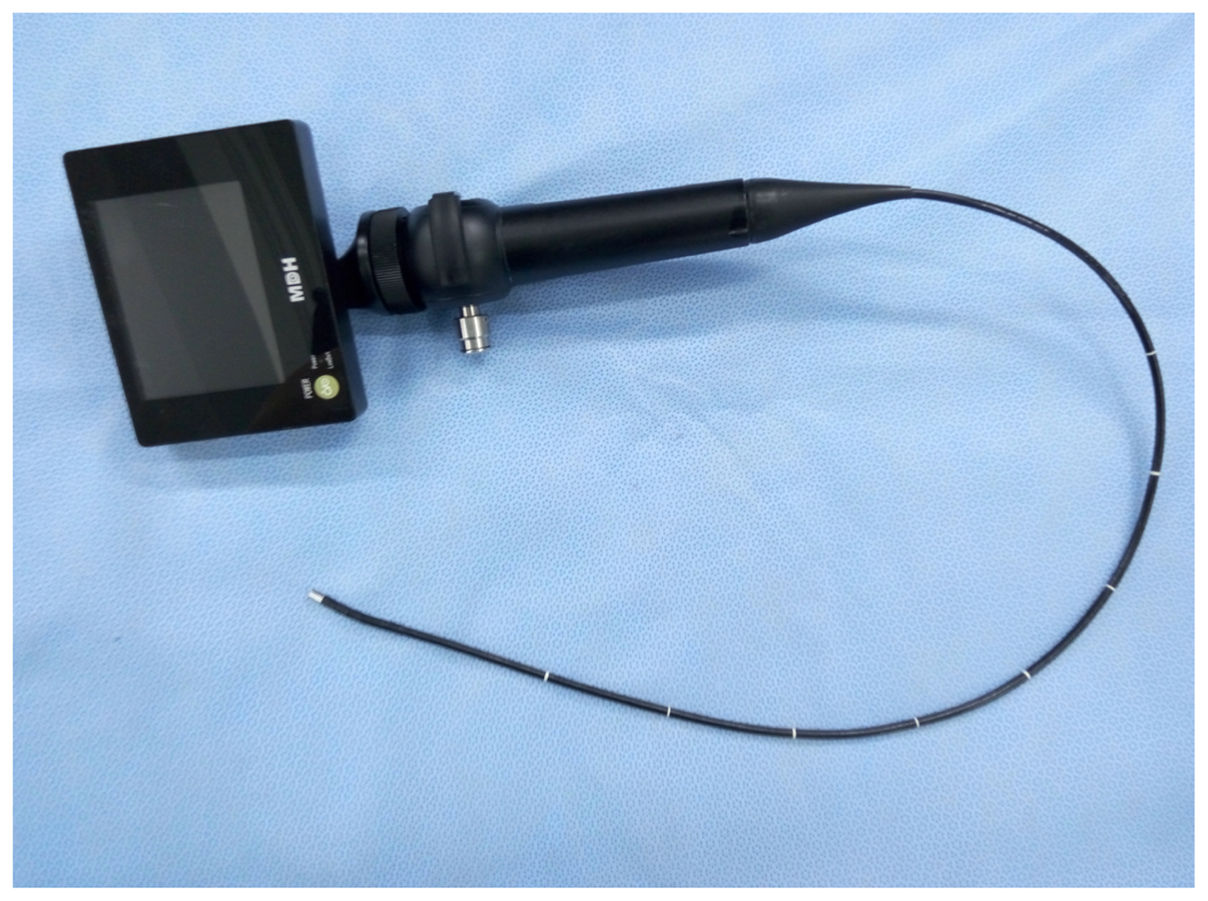
Visual intubation soft lens.

The flexible bronchoscope, the best instrument of airway management that was first used in Japan and America, has not been developed in early years in China. MDH Company and Youyi Company did not invent and manufacture different models of the visual flexible bronchoscope until the development of Cmos lens. Visual flexible bronchoscope may replace fiber bronchoscope in the clinic in the near future due to its advantages of portability, clear vision, and antifogging capabilities (Figure 20).

#### 1.11. Development of the airway auxiliary instrument

**Figure 21:**
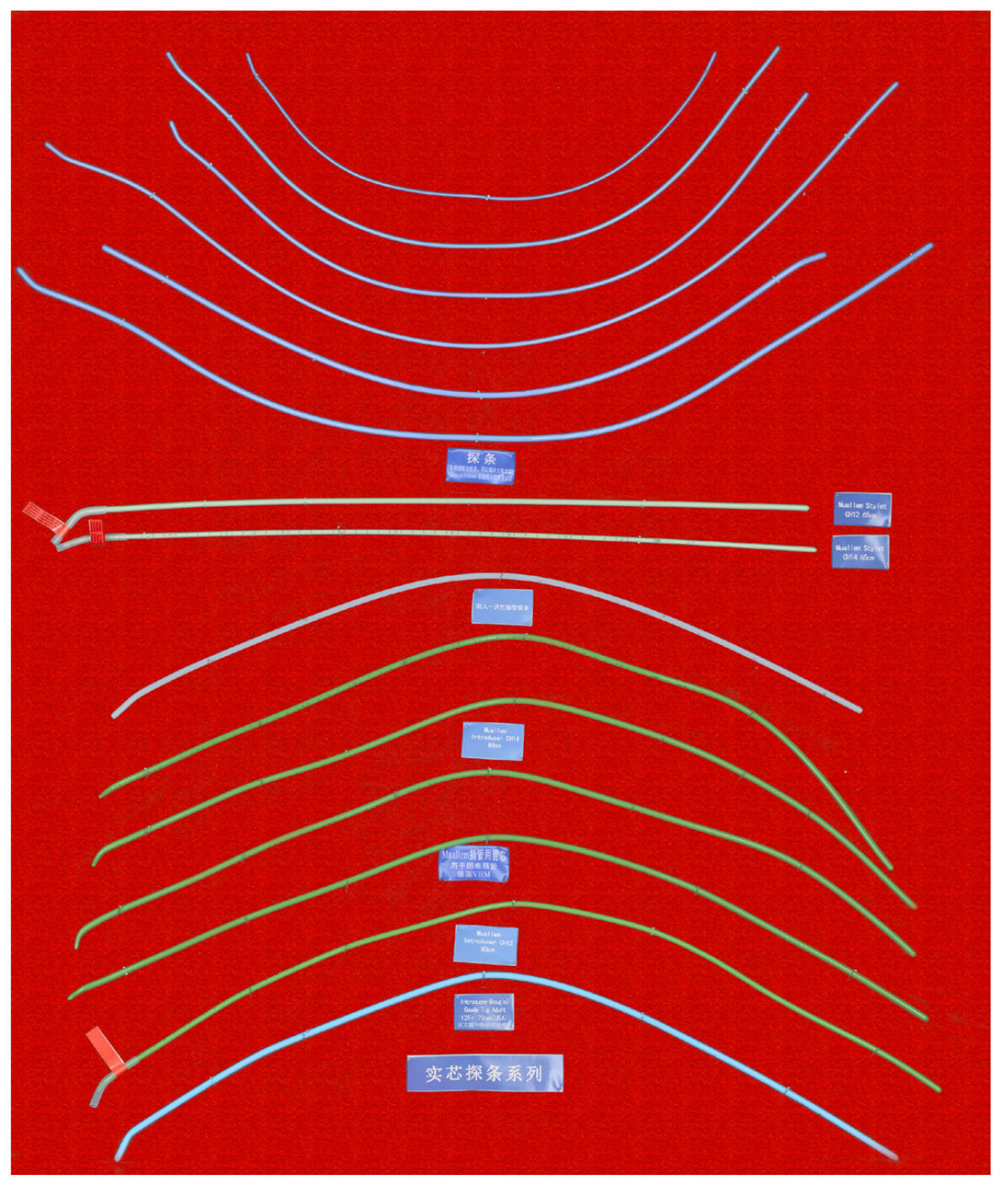
Traditional stylets.

The airway auxiliary instrument is an important element to airway management. Stylets and the tube exchanger are very important auxiliary instruments for airway management (Figure 21). Moreover, various kinds of the mouth gag, oropharyngeal airway and nasopharyngeal airway [17] play an important role in aiding airway management.

#### 1.12. Difficult airway cart [18]

Difficult airway cart is a novel facility in China that appeared in recent years; it is a comprehensive platform for emergent and difficult airway management and plays an important role in airway management. Difficult airway cart contains all the airway devices and airway auxiliary instruments, from noninvasive devices to invasive devices. There are also different kinds of medicines for emergent intubation.

### 2. The guide in developing difficult airway management in China

The knowledge of difficult airway management from USA [19, 20], France [21], Canada [22], England [23], Germany [24] and Italy [25] was the guide for the development of difficult airway management in China 20 years ago. The Chinese Society of Anesthesiology published the expert’s consensus of difficult airway management in the Journal of Clinical Anesthesiology in 2009 [26] and the procedure of difficult airway management in 2013 [27]. Professor Wu-Hua Ma proposed the “ABS” procedure, namely 1: Ask for help; 2: Breathe; 3: Spontaneous Breathing; 4: Stab cricothyroid membrane; 5): Surgical Airway. This procedure was published in the Journal of International Anesthesiology and Resuscitation [28] in 2013. It simplified the process of difficult airway management into four rules noted above. This sets a new developmental milestone of airway management in China.

### 3. Development of training of airway management in China

Many hospitals have paid special attention to the training of the management of difficult airway since the establishment of the guidelines of difficult airway management in China. These training programs have made considerable progress in changing airway management outcomes. There were different types of difficult airway management training workshops at different levels during the annual meeting of the Chinese Society of Anesthesiology; and there are also many bases for airway management training all over the country. Some bases have collaborations with world famous universities, such as Yale University and the University of Pennsylvania, to hold joint training classes.

### 4. Development of the research in airway management

There are nearly 1100 articles and 40 articles about airway instruments published in domestic and international journals respectively in recent 5 years. There is a substantial increase in the number of airway instruments available in practice and more new airway instruments have been invented and manufactured in China in recent years. Professor Jie Yi, Professor Yu-guang Huang and Professor Ai-lun Luo have investigated the current status of airway management in some provinces and cities in China, and Professor Wu-hua Ma has investigated the current status of airway management in Guangdong province. Data from the above investigations was published in the Journal of Clinical Anesthesiology and International Journal of Anesthesiology and Resuscitation.[29–31] These studies indicated that there are different choices of devices and methods for anesthesia intubation in China, more Chinese anesthesiologists would attempt alternative tools to intubate if they have difficult airway, and there are large variations between teaching and non-teaching hospitals in the knowledge and usage for other airway tools including emergency tools.

### 5. Establishment of the museum of airway instrument

**Figure 22:**
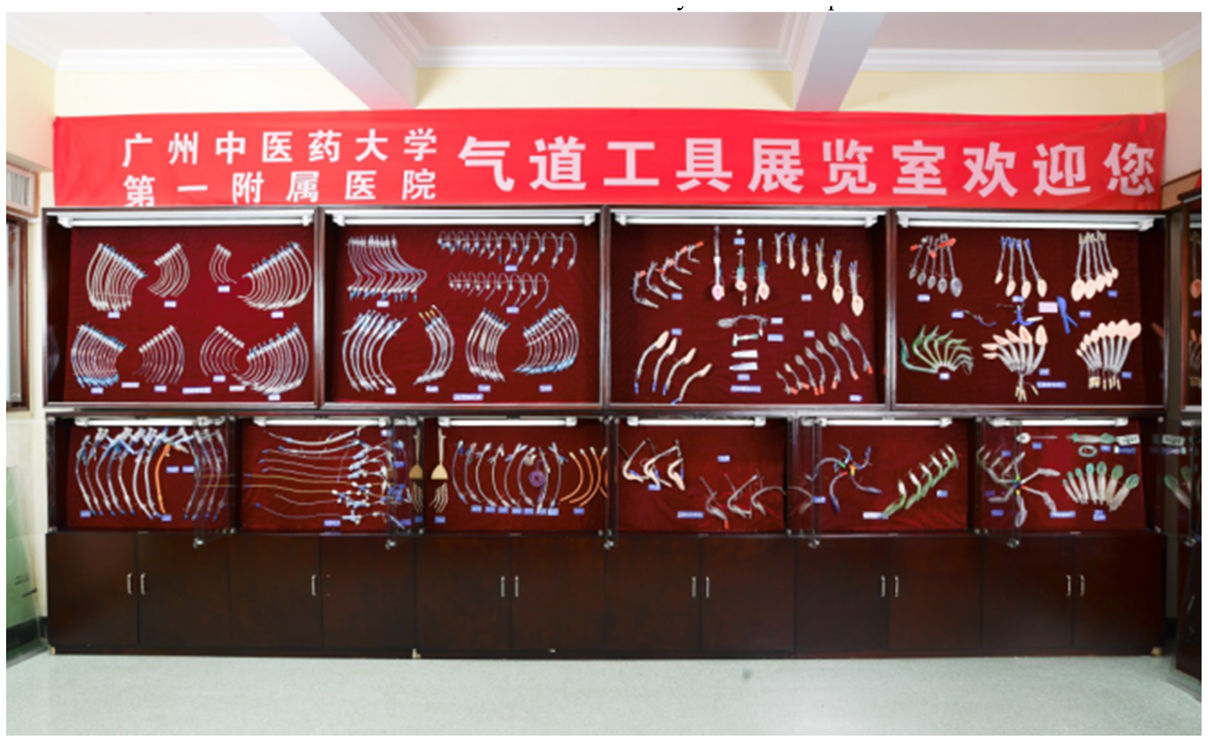
The museum of airway instruments

To help the young anesthesiologists gain a better understanding of the development of the airway management facilities and broaden the mind about its clinical application, Guangzhou University of Chinese Medicine established the first museum of airway instruments (Figure 22) in China. It has attracted many visiting experts from other countries.

## Conclusion

The airway is one of the most important systems during anesthesia and many other clinical situations related to oxygenation. However, it is very difficult to tackle without proper instruction by anesthesiologists and other airway experts. Scientific experts and doctors devote a lot of time to invent the ideal airway device to ensure safe ventilation for patients who undergo anesthesia or other therapy.

China, as a developing country, learned the advanced technology from the Western countries and introduced the airway management facilities into clinical practice in the earlier days. In recently years, Chinese experts made important contribution in improving the airway safety by developing novel airway devises. Chinese physicians will continue to play an important role in the development of safe airway management in the future.
